# Diagnostic accuracy of sperm DNA fragmentation index in male infertility: A cohort study

**DOI:** 10.5937/jomb0-59605

**Published:** 2026-01-06

**Authors:** Juan Zhang, Wei He

**Affiliations:** 1 Jiuquan Branch of Shanghai First People's Hospital, Department of Reproductive Medicine, Jiuquan, Gansu, 735000, China

**Keywords:** male infertility, sperm DNA fragmentation index, semen parameters, diagnosis, reproductive prognosis, muška neplodnost, indeks fragmentacije DNK spermatozoida, parametri sperme, dijagnoza, reproduktivna prognoza

## Abstract

**Background:**

To explore the correlation of sperm DNA fragmentation index (DFI) with semen quality, while assessing the diagnostic potential of DFI in male infertility, aiming to offer a novel biomarker and clinical approach for male fertility evaluation.

**Methods:**

A cohort of 613 men who visited our hospital between April 2023 and February 2025 was included in this study. Semen analysis (assessing concentration, motility, morphology, etc.) and sperm chromatin dispersion testing were conducted to determine DFI. The diagnostic performance of DFI for infertility was evaluated using receiver operating characteristic (ROC) curve analysis. Subgroup analyses (oligozoospermia, asthenozoospermia, and oligoasthenozoospermia) were conducted to assess the discriminative power of DFI. After treatment, the patients were followed up for 1 year, and the predictive effect of DFI on the prognosis of successful fertility was analysed.

**Results:**

Among infertile men (n = 92, incidence rate: 15.01%), DFI levels were significantly elevated compared to those with normal fertility (P&lt; 0.05). ROC curve analysis demonstrated that DFI had a sensitivity of 60.87% and specificity of 84.07% (AUC= 0.774) in diagnosing infertility. Notably, DFI displayed the highest discriminative efficacy for oligoasthenozoospermia (AUC= 0.825). Finally, the sensitivity and specificity of DFI for predicting successful fertility in infertile men at 1 year were 83.08% and 62.96%, respectively (P&lt; 0.001).

**Conclusions:**

DFI demonstrates high diagnostic accuracy on the fertility of infertile men and has a high clinical potential.

## Abstract (sr)

**Uvod:**

Cilj je bio da se ispita korelacija indeksa fragmentacije DNK spermatozoida (DFI) sa kvalitetom sperme, uz procenu dijagnostičkog potencijala DFI u muškoj neplodnosti, sa ciljem da se ponudi novi biomarker i klinički pristup za procenu muške plodnosti.

**Metode:**

U ovu studiju je uključeno 613 muškaraca koji su posetili našu bolnicu u periodu od aprila 2023. do februara 2025. godine. Analiza sperme (uključujući koncentraciju, pokretljivost, morfologiju itd.) i test disperzije hromatina spermatozoida su korišćeni za određivanje DFI. Dijagnostička vrednost DFI za infertilitet je procenjena analizom ROC (Receiver Operating Characteristic) krive. Sprovedene su i analize podgrupa (oligozoospermija, astenozoospermija i oligoastenozoospermija) radi procene diskriminativne moći DFI. Nakon terapije, pacijenti su praćeni godinu dana, a analiziran je i prediktivni značaj DFI za uspešnu plodnost.

**Rezultati:**

Kod neplodnih muškaraca (n = 92, učestalost: 15,01%) nivo DFI je bio značajno viši u poređenju sa muškarcima normalne plodnosti (P&lt; 0,05). ROC analiza je pokazala da DFI ima senzitivnost od 60,87% i specifičnost od 84,07% (AUC = 0,774) u dijagnostici neplodnosti. Posebno je DFI pokazao najvišu diskriminativnu efikasnost kod oligoastenozoospermije (AUC= 0,825). Konačno, senzitivnost i specifičnost DFI za predikciju uspešne plodnosti kod neplodnih muškaraca nakon jedne godine su iznosile 83,08% i 62,96% (P&lt; 0,001).

**Zaključak:**

DFI pokazuje visoku dijagnostičku tačnost u proceni plodnosti kod neplodnih muškaraca i poseduje značajan klinički potencijal.

## Introduction

In recent years, the global prevalence of male infertility has exhibited a marked increase. World Health Organisation (WHO) data indicate that approximately 15% of reproductive-aged couples encounter fertility challenges, with male factors contributing to 40%-50% of these cases [1] [2]. While semen analysis remains the cornerstone for assessing male fertility, its clinical limitations have become increasingly evident. Notably, research reveals that 15%-30% of infertile men present with normal conventional semen parameters yet still experience fertility issues [3], underscoring the potential inadequacy of traditional diagnostic indicators in identifying underlying pathologies. In this context, the sperm deoxyribonucleic acid (DNA) fragmentation index (DFI), a critical biomarker of sperm genetic material integrity, has emerged as a focal point in male fertility research [4]. Growing evidence, including studies by Birowo P et al. [5], establishes a significant association between elevated sperm DNA damage and reduced natural conception rates, suboptimal outcomes in assisted reproductive technology (ART), and an elevated risk of early pregnancy loss.

Despite these controversies, our study innovatively integrates DFI with longitudinal fertility outcomes to address the unmet need for stratified diagnostic criteria in populations undergoing natural conception. While some studies demonstrate a significant negative correlation between sperm DNA damage and conventional semen parameters (including motility and morphology) [6], others suggest a more complex, nonlinear relationship that may be modulated by multiple pathogenic mechanisms such as oxidative stress, abnormal apoptosis, and environmental factors [7]. Furthermore, existing investigations have predominantly focused on DFI's predictive value for ART outcomes [8] [9], with insufficient systematic analysis of its stratified assessment significance in natural conception populations. Notably, there is a paucity of comparative studies examining DFI variations among men with different fertility statuses. This theoretical gap has led to ongoing debates regarding the appropriate clinical applications and interpretation standards for DFI testing, significantly impeding its translation from research to clinical practice.

To address these limitations, our study represents the first comprehensive effort to integrate conventional semen analysis, DFI assessment, and longitudinal data on fertility outcomes. We place particular emphasis on elucidating the diagnostic efficacy of DFI in evaluating male fertility. Our research aims to uncover both the independent contribution and interactive mechanisms of DFI in assessing male fertility. The findings are expected to offer novel insights for male fertility evaluation and innovative approaches to reproductive health management [10].

## Materials and methods

### Research participants

This study included men who underwent semen analysis at our hospital between April 2023 and February 2025. The required sample size was calculated a priori using G*Power 3.1.9.2 (one-tailed test, effect size=0.10, α = 0.05, power=0.75), yielding a minimum of 534 subjects. After applying the inclusion and exclusion criteria, a final cohort of 613 participants was enrolled. The study protocol was approved by the Institutional Ethics Committee of our hospital, and all participants provided written informed consent.

### Inclusion and exclusion criteria

Inclusion criteria: Availability of routine semen analysis and sperm DFI data; Age 18 years; Married; Complete clinical records; Normal development of internal and external genitalia; Having fertility needs; The reproductive function of the partners was normal. Exclusion criteria: Severe oligozoospermia or azoospermia; Clinically significant varicocele or ejaculatory dysfunction; History of genital trauma/surgery; Family history of genetic disorders (e.g., Y chromosome microdeletions); Active urogenital infections, malignancies, or systemic diseases (e.g., hypertension, diabetes).

### Semen routine analysis

The patient was advised to refrain from sexual activity for 2 to 7 days prior to semen collection. The sample was obtained through masturbation into a sterile container. Following collection, the semen volume was measured, and the sample was incubated in a constant-temperature incubator at 37°C. Once complete liquefaction was achieved, 5 μL of semen was aspirated using a pipette and placed into a sperm counting chamber. An automated sperm quality analyser was then employed to assess key parameters, including semen concentration, total sperm count, sperm motility rate, and progressive motility (PR). Diagnostic criteria for male infertility [10]: Oligozoospermia i s defined as having PR 32%, along with either a total sperm count below 39x10^6^ or a semen concentration less than 15x10^6^/mL; asthenozoospermia is characterised by PR<32%, with a total sperm count exceeding 39x10^6^ and a semen concentration above 15x10^6^/mL; oligoasthenozoospermia is diagnosed when PR is below 32%, combined with either a total sperm count under 39x106 or a semen concentration less than 15x10^6^/mL. A fertile male meets all the following criteria: semen volume 1.5 mL, semen concentration 15x10^6^/mL, total sperm count >39x10^6^, sperm motility rate 40%, and PR 32%.

### DFI testing

Sperm DNA fragmentation was assessed using the sperm chromatin dispersion test. Semen slide preparation and staining procedures were performed in strict accordance with the manufacturer's protocol (Sperm DNA Fragmentation Detection Kit, Shenzhen BRED Life Science Technology Inc.). Semen samples were left at room temperature for 30 min for complete liquefication and stored in a water bath at 37°C for later use. 10 μL of liquefied semen was mixed with 190 μL of hypotonic buffer and incubated at 37°C for 30 min to promote sperm membrane permeability. 20 μL of treated semen was sucked and added to the preheated acid denaturation solution (pH 1.2) for 7 min to induce nucleoprotein depolymerisation. 200 mL of lysate containing SDS and DTT was added, and the sample was incubated for 5 min at room temperature to remove cell membrane and nuclear proteins. The reaction was terminated by washing the slide three times with PBS buffer. A 5 μL fluorescent dye mixture (acridine orange/DAPI) was then dropped onto the slide, covered with a cover glass, and incubated in the dark for 10 minutes. The tablets were sealed using an anti-fade sealer and stored at 4°C in the dark. An automated sperm quality analyser was used to randomly evaluate multiple fields of view, with a minimum of 500 spermatozoa counted and classified based on the presence and size of their halos. The ImageJ plug-in (DFI Calculator v1.2) was used to automatically calculate the DFI values. The quality control samples (concentration gradient: 5%/15%/30% DFI) provided by the BRED company were used to test the same quality control sample for 7 consecutive days before daily testing, and the CV value was <5%.

### Sex hormonal analysis

Fasting venous blood samples (3-5 mL) were collected from patients, and serum was separated via centrifugation. Sex hormone levels - including follicle-stimulating hormone (FSH), luteinising hormone (LH), prolactin (PRL), and testosterone (T) - were quantified using a fully automated chemiluminescence immunoanalyzer (Roche Cobas e601). Sample volume: 100 mL serum per well. Reaction conditions: Incubation at 37 °C for 18 min followed by double-antibody sandwich assay. Data reading: The instrument automatically calculates the concentration and generates a report. Internal quality control: Quality control was performed daily, both before, during, and after the test, and Westgard multi-rule correction was initiated when the results were out of control.

### Clinical treatment

After completing all reproductive function tests and identifying the aetiology, all patients received targeted treatment as advised by the doctor, such as gonadotropins and dopamine receptor agonists. In addition, vitamin E (400 IU/d), L-carnitine (2 g/d), and Coenzyme Q10 (200 mg/d) were administered to support spermatogenesis. For patients with genital tract infections, antibiotics (such as levofloxacin and cephalosporins) are typically used for 4-6 weeks, and a-receptor blockers (such as tamsulosin) are employed to improve the local microenvironment. The process of drug treatment is 3-6 months. If they are still unable to bear children after 6 months, assisted reproductive technology (ART), such as artificial insemination and a test-tube baby, can be considered.

### Prognosis, follow-up

All patients underwent a 1-year follow-up to assess prognosis, which was conducted through regular reviews and telephone follow-ups. The main follow-up index was whether the patient had a successful pregnancy (the partner had a successful pregnancy and did not have an abortion by the end of follow-up, was considered a successful pregnancy, 12 gestational weeks and live birth).

### Study parameters

The diagnostic efficacy of DFI in identifying male infertility and distinguishing between infertility subtypes was evaluated. Additionally, the correlation between DFI and semen quality parameters, as well as sex hormone levels, was investigated.

### Statistical analysis

Statistical analyses were conducted using SPSS 24.0 (IBM Corp.). Categorical variables, presented as frequencies and percentages [n (%)], were analysed using χ^2^ tests. Continuous variables, expressed as mean ± standard deviation (χ̄±s), were verified for normal distribution through Shapiro-Wilk testing before analysis [normality was tested using the Shapiro-Wilk test (significance level α = 0.05). Variables with *P*<0.05 were considered non-normally distributed. Normally distributed continuous variables were compared using independent two-sample t-tests. Pearson's correlation analysis was employed to examine relationships between variables. Receiver operating characteristic (ROC) curve analysis was performed to evaluate diagnostic performance, with the area under the curve (AUC) serving as an indicator of predictive accuracy (the closer the AUC is to 1, the better the performance). The cut-off value, sensitivity and specificity were determined according to the maximum Youden index. Statistical significance was defined as a two-tailed *P*-value less than 0.05.

## Results

### Semen analysis findings

Among the 613 men evaluated, 92 (15.01%) were diagnosed with infertility, a prevalence consistent with previously reported rates in the literature [11]. The infertility cases comprised 38 men with oligozoospermia, 32 with asthenozoospermia, and 22 with oligoasthenozoospermia. Comparison of clinical baseline data between infertile and normal men revealed that none of the differences between the groups were statistically significant (*P*>0.05). However, FSH, LH, and PRL were higher in infertile men than in normal men, while T was lower than in normal men (*P*<0.001), Table 1.

**Table 1:** Comparison of baseline information.

	Normal men (n = 512)	Infertile men (n = 92)	t (or χ^2^)	*P*
Age	31.04±2.94	31.50±3.11	1.375	0.170
BMI	22.89±1.96	22.58±3.18	1.256	0.210
Abstinence time (d)			2.307	0.129
2-4	184 (35.32)	25 (27.17)		
>4-7	337 (64.68)	67 (72.83)		
Smoking	342 (65.64)	68 (73.91)	2.414	0.120
Drinking	226 (43.38)	48 (52.17)	2.447	0.118
Exercise habits			2.971	0.085
Yes	184 (35.32)	24 (26.09)		
No	337 (64.68)	68 (73.91)		
Fertility history			2.194	0.139
Yes	82 (15.74)	9 (9.78)		
No	439 (84.26)	83 (90.22)		
FSH (mIU/mL)	6.28±3.72	9.52±4.70	7.383	<0.001
LH (mIU/mL)	4.17±2.00	6.36±2.68	9.182	<0.001
PRL (ng/mL)	10.24±6.02	16.92±6.97	9.566	<0.001
T (ng/mL)	4.40±2.60	3.42±1.94	3.463	<0.001

### Diagnostic performance of DFI in infertility

**Figure 1:**
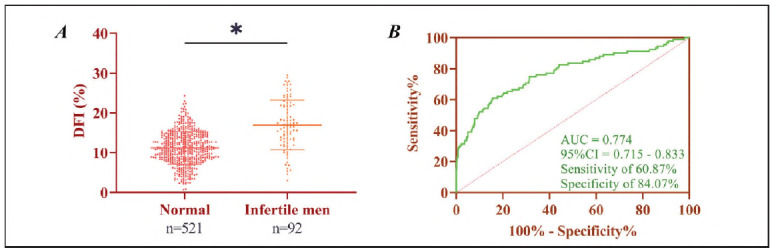
Role of DFI in male infertility.<br>A) Comparison of DFI in infertile and normal men. B) ROC curve of DFI for diagnosis of male infertility. *P < 0.05.

DFI was significantly elevated in infertile men compared to those with normal fertility (11.22±4.15% vs. 16.95±6.26%, *P*<0.05). ROC curve analysis identified a DFI cut-off value of greater than 15.25% as diagnostically significant, with a sensitivity of 60.87% and specificity of 84.07% for detecting infertility (AUC = 0.774, *P*<0.05), as shown in Figure 1.

### Subgroup analysis

**Figure 2:**
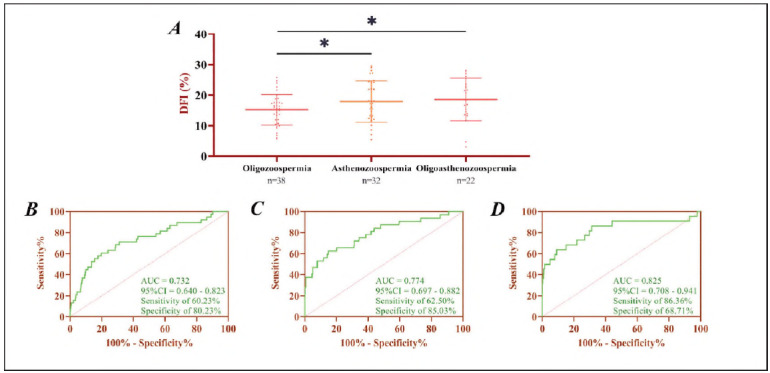
Relationship between DFI and oligozoospermia, asthenozoospermia, and oligoasthenozoospermia.<br>A) Comparison of DFI in oligozoospermia, asthenozoospermia, and oligoasthenozoospermia patients. B) ROC curve of DFI for diagnosis of asthenozoospermia. C) ROC curve of DFI for diagnosis of oligozoospermia. D) ROC curve of DFI for diagnosis of oligoasthenozoospermia. *P < 0.05.

Further subgroup analysis of DIF in patients with oligozoospermia, asthenozoospermia, and oligoasthenozoospermia revealed that there was no significant difference in DFI observed between asthenozoospermia and oligoasthenozoospermia groups (*P*>0.05). Still, both were higher than those with oligozoospermia (*P*<0.05). Subsequently, ROC curve analysis was conducted using DIF values from patients with oligozoospermia (Cut-off >14.81%, AUC=0.790), asthenozoospermia (Cut-off >15.42%, AUC=0.732), and oligoasthenozoospermia (Cut-off >13.20%, AUC = 0.825), as well as normal male controls. The results demonstrated that DIF had strong discriminatory power in distinguishing these conditions, with the highest diagnostic accuracy observed for oligoasthenozoospermia, (Figure 2).

### Predictive effect of DFI on successful fertility in infertile men after treatment

**Figure 3:**
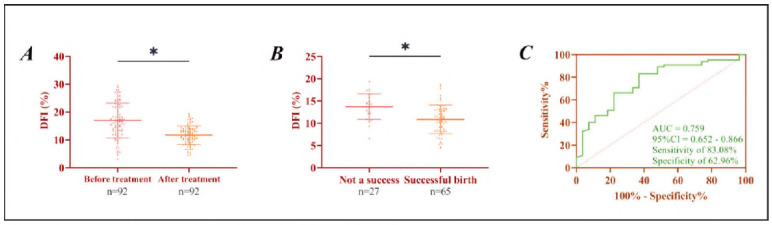
Analysis of prognostic significance of DFI in infertility.<br>A) Comparison of DFI before and after treatment in infertile men. B) Comparison of DFI between patients with and without prognos- tically successful childbearing. C) ROC curve of DFI for predicting successful fertility in infertile men after treatment. *P < 0.05.

After treatment, the DFI of all patients was significantly lower than before treatment (*P*<0.05). Subsequently, during the one-year follow-up survey, a total of 27 (29.35%) patients successfully gave birth. The DFI of patients who experienced successful childbearing after treatment was lower than that of patients who did not (*P*<0.05). The ROC curve showed that DFI<13.37% had a sensitivity of 83.08% and a specificity of 62.96% (*P*<0.001) for predicting successful fertility within 1 year after treatment (Figure 3).

## Discussion

Male infertility is the main factor leading to about 40-50% of couples being unable to conceive [12]. This study demonstrates that the sperm DFI exhibits excellent diagnostic and discriminative efficacy for male infertility. Our findings offer valuable insights for infertility assessment and have the potential to optimise current diagnostic and therapeutic approaches in clinical practice.

First, we observed significantly higher DFI levels in infertile men compared to those with proven fertility. These findings align with existing literature while providing novel perspectives. The underlying mechanisms may involve oxidative stress, which has been identified as a primary contributor to sperm DNA damage. As highlighted by Takalani NB et al. [13], excessive reactive oxygen species (ROS) can induce nuclear DNA damage, destabilise chromatin structure, impair mitochondrial function, and consequently reduce sperm motility and concentration. Moreover, aberrant apoptosis may further exacerbate DNA fragmentation. Particularly when the regulation of spermatogenic cell apoptosis is disrupted, immature sperm are prematurely released into the semen, leading to a decline in semen quality [14]. Notably, while some studies report nonlinear relationships between DFI and semen parameters - potentially attributable to environmental influences or epigenetic modifications [15] - our results reveal a robust correlation after stringent adjustment for confounders (e.g., severe varicocele, infections). This consistency underscores DFI as an independent biomarker of impaired sperm function, reinforcing its clinical utility in male infertility evaluation.

To validate our perspective, we evaluated the diagnostic efficacy of DFI in male infertility using ROC curve analysis. The results demonstrated that DFI achieved a sensitivity of 60.87% and a specificity of 84.07% (AUC=0.774) in diagnosing infertility, indicating its potential clinical utility as an auxiliary diagnostic tool. Furthermore, subgroup analysis revealed particularly outstanding discriminatory efficacy of DFI for oligoasthenozoospermia cases (AUC = 0.825), suggesting its superior diagnostic performance in this specific patient population. These findings are consistent with the research by Liu et al. [16], which reported that DFI's predictive value for ART outcomes significantly surpassed that of conventional semen parameters. Our study further expands the clinical applications of DFI, highlighting its stratified diagnostic significance in natural fertility populations. For instance, clinical observations indicate that approximately 15%-30% of infertile men exhibit normal conventional semen parameters. Yet, elevated DFI levels may uncover underlying genetic damage [17], thereby compensating for the limitations of traditional semen analysis. Additionally, DFI testing offers distinct advantages, including strong objectivity and high reproducibility. Standardised procedures, such as the sperm chromatin dispersion test, can minimise subjective interpretation errors [18], providing clinicians with a more precise and reliable diagnostic tool for assessing male infertility.

Finally, we found that the DFI of the patients after treatment was lower than that before treatment, again emphasising the close relationship between DFI and fertility in infertile patients. At the same time, DFI also demonstrated a positive impact on the prognosis of successful fertility through the follow-up survey, emphasising the potential importance of DFI as an indicator of male fertility in the future. However, it is worth noting that some patients in the follow-up survey had successful childbearing through ART. As we all know, the success of ART primarily depends on the operator's professional technology [19] [20]. Our analysis included such patients, and the results may not be fully representative of the effectiveness of DFI in assessing fertility. This is because we were unable to exclude these patients from subgroup analyses due to the small number of cases. Therefore, we need to carry out more in-depth and comprehensive research as soon as possible to verify the effect of DFI in the evaluation of the prognosis of successful fertility in infertile patients.

Based on the findings of this study, we recommend that DFI testing should be prioritised for men with normal semen parameters but persistent infertility to assess potential genetic material damage. According to the DFI results, clinicians can implement targeted interventions, including antioxidant therapy (e.g., vitamin C and coenzyme Q10 supplementation) or lifestyle modifications (e.g., smoking cessation, weight management), to mitigate oxidative stress. Furthermore, integrating sex hormone profiling with DFI testing may facilitate the development of personalised therapeutic strategies for male infertility patients, ultimately improving clinical outcomes.

Nevertheless, this study has several limitations. First, the single-centre design may restrict the generalizability of our conclusions. Second, while we utilised the sperm chromatin dispersion test for DFI assessment, we did not evaluate its concordance with other established techniques such as TUNEL or SCSA. Third, the relatively short follow-up period precluded the evaluation of long-term reproductive outcomes, thereby limiting our ability to validate DFI's predictive value for natural conception rates. Future research should address these limitations through multicenter, large-scale cohort studies encompassing diverse geographic and ethnic populations. Additionally, comparative studies evaluating different DFI detection methodologies are warranted to establish standardised diagnostic thresholds and optimise clinical utility.

## Conclusion

There is a close relationship between DFI and male fertility. DFI can effectively evaluate the occurrence of infertility and identify the types of infertility. At the same time, DFI can also provide a reference for the successful fertility of infertile men. These findings offer new reference and guidance for the diagnosis and treatment of male infertility in the future. However, the concordance of DFI values across different detection platforms (e.g., TUNEL, SCSA) remains unexplored, which warrants future methodological validation.

## Dodatak

### Consent to publish

All authors gave final approval of the version to be published.

### Availability of data and materials

The data used to support the findings of this study are available from the corresponding author upon request.

### Funding

No funding was received for conducting this study.

### Acknowledgements

Not applicable.

### Conflict of interest statement

All the authors declare that they have no conflict of interest in this work.
